# Exploring the implementation of an ICF-based instrument and guideline for interprofessional collaboration in the return-to-work process

**DOI:** 10.1177/10519815251409721

**Published:** 2026-01-09

**Authors:** Donny Kreuger, Bouwine Carlier, Birgit HPM Donker-Cools, Johannes R Anema, Frederieke G Schaafsma, Shirley Oomens

**Affiliations:** 1 Public and Occupational Health, Amsterdam UMC Location University of Amsterdam, Amsterdam, the Netherlands; 2 Societal Participation and Health, Amsterdam Public Health Research Institute, Amsterdam, the Netherlands; 3 Research Center for Insurance Medicine, Amsterdam, The Netherlands; 4 Occupation and Health Research group, Han University of Applied Sciences, Nijmegen, the Netherlands; 5 Radboudumc, Department of Primary and Community Care, Nijmegen School of Occupational Health, Nijmegen, the Netherlands

**Keywords:** return to work, sick leave, occupational health, international classification of functioning, disability, and health, implementation science, occupational health physicians

## Abstract

**Background:**

Interprofessional collaboration among occupational health professionals (OHPs) is essential for guiding sick-listed employees and facilitating return to work (RTW). However, the lack of a shared language among different OHPs can hinder effective collaboration. To address this, an instrument and multidisciplinary guideline based on the International Classification of Functioning, Disability, and Health (ICF) were developed.

**Objective:**

This study aimed to assess the feasibility of the ICF-based instrument and multidisciplinary guideline, as well as to explore OHPs’ experiences to support implementation in daily practice.

**Methods:**

A triangulated mixed-methods design was used, combining OHPs assessing work capacity with the instrument in practice for sick-listed employees, followed by interviews, case reviews, and focus groups with both medical and non-medical OHPs. The Measurement Instrument for Determinants of Implementation (MIDI) guided data collection and analysis.

**Results:**

OHPs experienced the ICF-based instrument as comprehensible, usable for providing sick leave guidance, and particularly valuable for enabling qualitative assessments of work capacity and RTW possibilities. The so-called d-codes included in the instrument, derived from the ICF-framework, facilitated communication between medical and non-medical professionals. OHPs also found the instrument supported shared decision-making by incorporating both employees’ and employers’ perspectives. Application was especially suited for complex cases of long-term sick leave.

**Conclusions:**

This study highlights the potential of implementing the ICF-based instrument and multidisciplinary guideline in occupational health practice to improve interprofessional collaboration during sick leave and RTW. OHPs reported that the instrument supports capturing both the strengths and limitations of sick-listed employees, while also addressing workplace and personal factors.

## Introduction

The return-to-work (RTW) process often involves different occupational health professionals (OHPs) whose collaboration is essential for facilitating employee rehabilitation, particularly in cases of long-term sick leave.^1–3^ Previous research highlights the importance of a coordinated return-to-work (RTW) process that integrates the expertise of different OHPs to ensure employees receive high-quality support, guidance, and timely occupational health services.^4^

Different OHPs often approach an employee's situation from separate perspectives, which can result in inconsistent assessments and conflicting recommendations during rehabilitation and RTW planning.^5–8^

Since these assessments and recommendations form the basis for RTW advice and sick leave guidance, such discrepancies can hinder effective interprofessional collaboration. A shared understanding of the various factors influencing employee rehabilitation is therefore essential for coordinated RTW support.^5–8^

The International Classification of Functioning, Disability, and Health (ICF), introduced by the World Health Organization (WHO), provides a valuable framework for this purpose, and is used to enhance collaboration by offering a biopsychosocial perspective on health and functioning, integrating medical, environmental, and personal factors.^9–15^ Studies increasingly emphasize the utility of the ICF-framework in capturing the multidimensional aspects of patient recovery and rehabilitation.^15–19^

The ICF-framework is particularly well-suited for RTW and sick leave guidance purposes, as it underlines a interactionist perspective, focusing on personal, social, and environmental factors which are important to consider for rehabilitation.^14,^^19–22^

An example of the utility of the ICF for understanding and supporting rehabilitation is Lervik's study on self-perceived RTW barriers, which found that individuals on long-term sick leave report obstacles across all ICF domains, especially in environmental factors (e.g., lack of support or systemic barriers) and activity limitations.^23^ Furthermore, other studies emphasize the importance of incorporating employees’ experiences during sick leave and RTW to provide tailored support.^7,24,25^

These findings highlight the need for instruments that capture the multifaceted nature of long-term sick leave, including the dynamic interaction of contributing factors and the employee's own RTW perspective. Although the ICF is explicitly designed to integrate medical, personal, and environmental aspects of functioning, instruments based on the ICF have often relied on quantitative scoring systems.^10–12^^,26,27^ This may limit their ability to capture the contextual nuances characteristic of complex long-term sick leave cases, which can inform personalized RTW strategies, and reflect the employee's lived experience. As a result, the ICF's potential as a tool for structured dialogue, contextual analysis, and shared understanding of RTW barriers remains underutilized in occupational health practice.

In a prior Delphi study the potential of the ICF classicisation system as a shared language for enhancing interprofessional collaboration among OHPs was investigated.^3^ Consensus was reached among 12 OHPs on 20 essential ICF-codes from the activities and participation (d) domain for assessing work capacity, along with items related to work- and personal factors. Based on these findings, a new ICF-based instrument (for the description of work capacity and RTW possibilities) and an accompanying multidisciplinary guideline were developed (see Appendix 1).

While the ICF itself is well-established in the field of rehabilitation, it is essentially a classification system used to describe a static situation of health and functioning. The novelty of our study lies in the feasibility testing of a new ICF-based instrument that dynamically captures work capacity and RTW possibilities over time. The instrument operationalizes the biopsychosocial model and translates the ICF classification system into a practical instrument that enables OHPs to promote sick leave guidance and RTW collaboration. The present study therefore aims to evaluate the feasibility of implementing this instrument and to explore OHPs’ experiences with its use in practice.

## Methods

**Figure 1.**
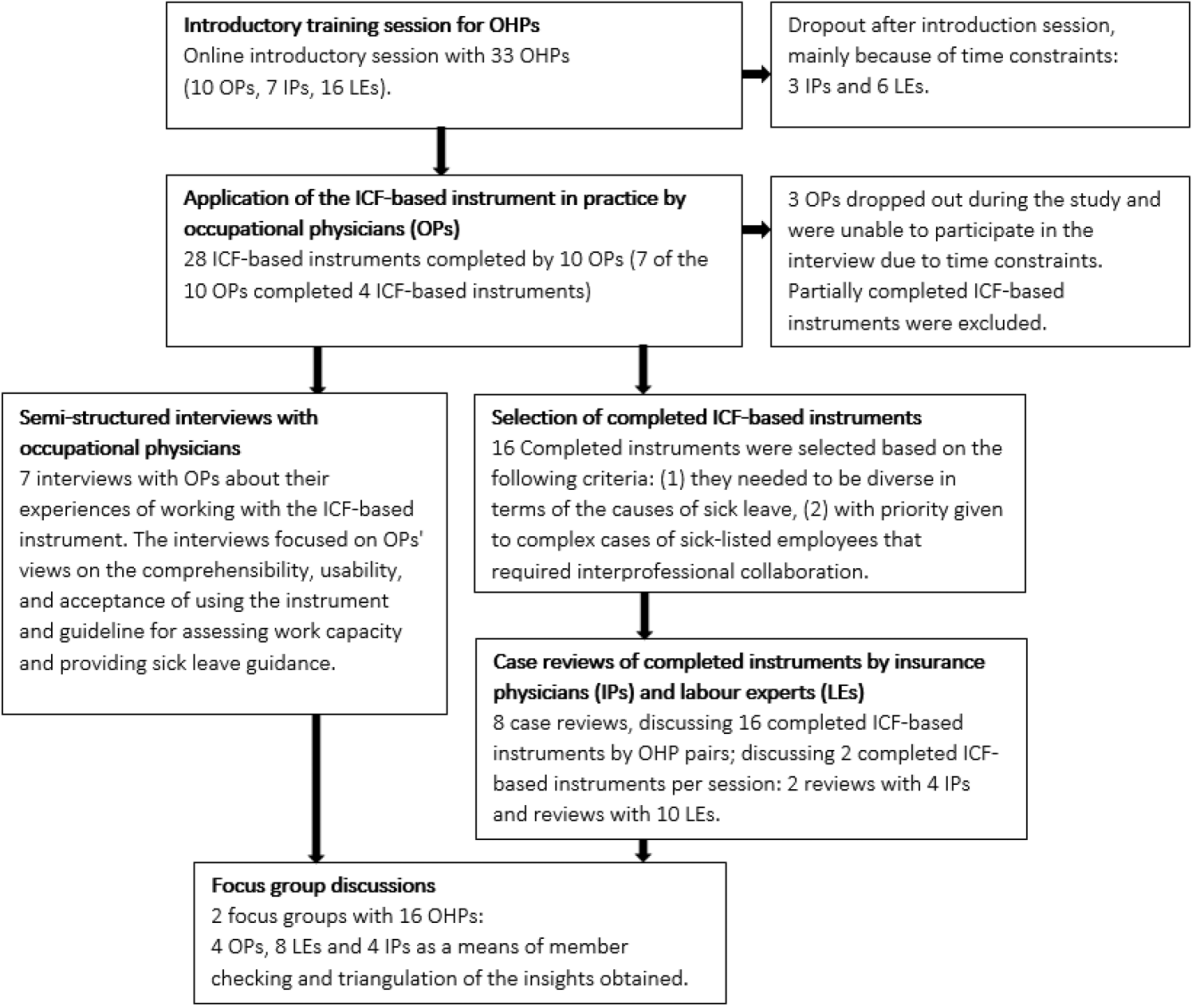
Flowchart of the study.

A triangulated mixed-methods design was employed to explore how OHPs from different professional backgrounds experienced both completing and interpreting the ICF-based instrument in daily practice (see Figure 1). This design enabled the collection of complementary data through (1) semi-structured interviews, (2) reviews of completed ICF-based instruments, and (3) a focus group discussion. The focus group was used to triangulate preliminary findings from the earlier phases to inform strategies for the implementation of the ICF-based instrument in occupational health practice. By comparing and integrating data across these methods and professional perspectives, the study aimed to enhance the validity of the findings.^28,29^

### RTW system in the Netherlands

In the Netherlands, the RTW process is governed by a legal framework that outlines the responsibilities of both employees and employers guided by OHPs, throughout a maximum duration of 2 years of sick leave.^8,26^ Different OHPs are involved in sick leave guidance and the RTW process, including occupational physicians (OPs), labour experts (LEs), and insurance physicians (IPs), all of whom play a central role in this process.^3^ These OHPs have different tasks and responsibilities, such as assessing employees’ work capacity, advising on RTW-interventions, and identifying potential work adaptations.^30^ During sick leave and the RTW process, OPs assess work capacity and RTW possibilities. LEs, who focus on workplace adaptions and IPs, who perform disability assessments interpret this information within the context of their respective roles.^3^ In this study, OPs applied the ICF-based instrument in practice to assess work capacity and to provide sick leave guidance. Subsequently, the completed instruments were anonymized and shared with LEs and IPs to explore whether they provided a clear understanding of work capacity and facilitated interprofessional collaboration.

### Determinants for implementation

The topics and questions used in the interviews with OPs, the case reviews of completed ICF-based instruments with LEs and IPs, and the focus groups with a selection of all OHPs were derived from the Measurement Instrument for Determinants of Innovations (MIDI).^31^ MIDI covers 29 determinants for implementing health innovations across the following domains: (1) the socio-political context, (2) the organisation, (3) the user/health professional and (4) the innovation itself. Fleuren et al. mention that determinants from the MIDI should be selected and tailored to the specific context of the innovation under study.^31^ Accordingly, determinants related to comprehensibility, usability, and acceptance were selected as the foundation for the interview guideline, case review questionnaire, and focus group guide (see Appendices 2, 3, and 4). The analytical framework for analysing the data encompassed all domains of the MIDI, as OHPs were free to bring up new topics.

### Participants and procedures

OHPs working actively in occupational healthcare settings were recruited through purposive sampling to ensure a balanced group of OPs, IPs, and LEs. These professional groups represent the key disciplines involved in the Dutch RTW system. Recruitment primarily took place through professional associations, including the NVAB (Dutch Association for Occupational Medicine), the NVVG (Dutch Association for Insurance Medicine), and NVva (Dutch Association for Labour Experts), to ensure participation of certified professionals with relevant expertise and experience. In addition, recruitment was extended via LinkedIn and the project website to reach OHPs who may not be affiliated with these associations but are actively engaged in RTW processes. The study did not aim to recruit a fixed number of OHPs but rather sought to ensure diversity in professional background.

### Ethics considerations & informed consent

The Ethical Commission of Research (ECO) of the HAN University of Applied Sciences assessed that ethical approval was not required for this study, *as they assessed and declared that the study was not subject to the Dutch Medical Research Involving Human Subjects Act (ECO 457.0623)*. Participants were informed about the study objectives during the online introductory session. Written informed consent for participating in the study was obtained via email prior to participation.

### Introductory session for OHPs

All participants were invited to an online introductory session, receiving an overview of the study objectives and procedures. Prior to the introduction, participants were asked to study the ICF-based instrument and multidisciplinary guideline thoroughly. During the introduction session, participants received instructions on how to use the ICF-based instrument and multidisciplinary guideline in their practice, and they were able to ask questions about the study procedures.

### Application of the ICF-based instrument in practice by occupational physicians (OPs)

Following the introductory session, OPs were instructed to complete the ICF-based instrument for four ongoing sick leave cases in their practice. While OPs had the flexibility to choose when to use the ICF-based instrument, they were encouraged to select cases of sick leave that varied in both complexity and duration. Once completed, the ICF-based instruments were sent via e-mail to the researchers. To ensure confidentiality, OPs anonymized personal information, and researchers verified that no identifiable details about the employee or employer were included.

### Semi-structured interviews with occupational physicians

OPs were invited for an online interview to discuss their experiences using the ICF-based instrument and multidisciplinary guideline in practice. The interviews focused on OPs’ views on the comprehensibility, usability, and acceptance of using the instrument and guideline for assessing work capacity and providing sick leave guidance (see Appendix 2). Interviews were audio recorded, and the researcher took notes during the interview. A selection of the completed ICF-based instruments by OPs were used for the case reviews with LEs and IPs.

### Selection of completed ICF-based instruments

Completed instruments by OPs were selected based on the following criteria: (1) they needed to be diverse in terms of the causes of sick leave, (2) with priority given to complex cases of sick-listed employees that exemplify real-life scenarios requiring interprofessional collaboration.

IPs and LEs each received two completed ICF-based instruments by e-mail, along with a questionnaire (Appendix 3). IPs and LEs individually reviewed the completed instruments and subsequently completed a questionnaire focused on whether the instrument provided a clear interpretation of work capacity and to what extent it supports interprofessional collaboration.

### Review of completed instruments by insurance physicians (IPs) and labour experts (LEs)

Either two LEs or two IPs were randomly paired by the researchers for an online case review.

The goal of the case reviews was to evaluate the consistency of interpretations of work capacity among OHPs from the same profession for completed ICF-based instruments. During the review, OHP pairs used their completed questionnaires to compare interpretations of work capacity and discussed the feasibility of the instrument and guideline to support interprofessional collaboration. At least one researcher was present during these meetings to moderate the case review and ask additional questions. The case reviews were audio recorded, and the researcher took notes of the discussions.

### Focus group discussions

OHPs who participated in the interviews and case reviews were invited to a focus group to reflect on the obtained insights and to discuss how the ICF-based instrument can support interprofessional collaboration (see Appendix 4 for guideline). A summary of the insights from the interviews and case reviews was sent to the OHPs, serving as a means of member checking the findings with the participants. The focus groups were audio recorded, and the researchers took notes during the discussions.

### Data analysis

The interviews with OPs and the focus groups were transcribed verbatim. Data analysis was performed in MAXQDA 2022, focussing on identifying themes related to OHPs’ experiences with the comprehensibility, usability, and satisfaction of the ICF-based instrument and multidisciplinary guideline. A deductive coding scheme was developed based on the MIDI domains as analytical framework. The following topics formed the basis for the deductive coding scheme:

(1) MIDI determinants of the innovation, (2) MIDI determinants of the organization, (3) MIDI determinants of the user, (4) MIDI determinants of the socio-political context, (5) experiences of collaborating with other OHPs/stakeholders using the instrument and guideline, (see Appendix 5 for final coding scheme).

The qualitative data analysis was guided by the steps of thematic data analysis.^32,33^ The analytical process involved researchers DK and BC reading the transcripts of the interviews and focus groups for familiarisation. The same researchers independently coded two transcripts. Any disagreements regarding the codes were discussed until consensus was reached. Sub-codes were added to the deductive coding scheme when new topics emerged. The remaining transcripts were coded by DK, and coded segments were exported to Microsoft Excel for further analysis of overarching themes. Researchers DK, SO, BC, BDC and FS discussed the themes that emerged from the analysis for further validation.

## Results

In total 21 OHPs (7 OPs, 4 IPs and 10 LEs) participated in the study, of whom 11 were female and 10 were male participants. Between May and November 2023, ten OPs completed a total of 28 ICF-based instruments, seven OPs were interviewed. Sixteen completed ICF-based instruments were selected for case reviews by ten LEs and four IPs. The findings from the interviews and case reviews were discussed in two focus groups, each consisting of eight OHPs, for a total of 16 participants (see Figure 1).

### Determinants of the user

#### Using the ICF-based instrument for interprofessional collaboration

The ICF-based instrument was applied at various stages of sick leave and the RTW process, including the initial stage (N = 7), one year after the onset of sick leave (N = 18), and before employees applied for disability benefits after two years of sick leave (N = 4). Most OPs completed the ICF-based instrument approximately one year after the onset of sick leave, in accordance with Dutch legislation. At this point, collaboration with non-medical OHPs is required to assess whether an employee can return to their original job.

Completing the ICF-based instrument took OPs between 15 and 60 min and depended on case complexity and the professional's familiarity with the instrument. OPs reported that cases involving multi-morbidity, fluctuating health conditions, or subjective health complaints were more complex to assess. Complexity also increased when workplace adaptations were needed, when psychosocial factors (e.g., coping, motivation, or family situation) played a role, or when expectations between employee and employer differed. These factors are typical of long-term sick leave. OPs noted that the ICF-based instrument helps capture such complexities, identify tailored solutions, and facilitate interprofessional collaboration. Although more time is needed in these cases, they emphasized its value for comprehensive assessment and effective guidance toward RTW.

#### Comprehensive description of workplace factors

The completed ICF-based instrument provided non-medical OHPs with detailed insights into the RTW trajectory, job activities, and workplace conditions affecting the RTW process. This allowed them to propose alternative work arrangements and workplace adjustments. OHPs found the instrument usable for developing tailored RTW plans, as it effectively addressed challenges unique to each employee's personal and work context. Moreover, OHPs stressed that the d-codes of the activities and participation domain of the ICF enabled collaboration between medical and non-medical OHPs.

“This is how I use it with employees as well. When I talk to them, they often say, ‘I’m sick and have these issues.’ I explain that this instrument provides a translation, a way to describe how their condition relates to their specific work environment and context. It's not about the illness itself, but how it impacts their ability to work. I find this approach more accessible than just presenting a list of limitations. The simpler language helps employees understand it more easily.” (Quote from participant 3).

### Determinants of the innovation

#### Current and future RTW possibilities

OHPs highlighted the ICF-based instrument's emphasis on RTW possibilities as a key advantage which supports more effective guidance of employees and employers. OHPs noted that the instrument allows them to describe both current and anticipated future RTW possibilities. Moreover, asides from only describing challenges and limitations, OHPs mentioned that the ICF-based instrument can be used to describe individual strengths which can integrated to RTW advise. According to OHPs, this aligns with the concept of positive health. Additionally, they highlighted the usability of the ICF-based instrument in outlining conditions that can effectively support and guide the RTW process. According to OHPs, this improves the instrument's usability by helping employees and employers better understand and effectively apply the information and advice provided. A participant noted during the focus group:

“the instrument allows me to describe what is currently possible and what is expected to be possible in the future. For an employee, it's particularly important to understand how they can return to work and the ICF-based instrument can be used to support that” (Quote from participant 3).

#### Understanding the employee's point of view

OHPs particularly appreciated that the ICF-based instrument enabled them to describe the employees’ understanding of their own work capacity and RTW possibilities, as well as the impact of these on both work and daily life. They noted that the instrument captures work capacity and RTW possibilities from the employee's perspective, helping OHPs understand the employee's experiences and develop tailored solutions to challenges during the RTW process. A participant mentioned the following during a case review:

“if someone experiences discomfort during an activity or movement but can still perform the task, the question isn't so much about their functional limitations, like “How much can they physically handle?”

The real question is, “To what extent are they willing to perform the activity?” This doesn't reflect the severity of the complaint, it reveals valuable information about personal characteristics, such as their coping mechanisms. It indicates whether the person is willing to endure the discomfort because they enjoy what they are doing or because they feel it's worth it” (Quote from participant 5).

#### A qualitative description of work capacity

Most OHPs found the quantification of work capacity in the ICF-based instrument not usable, noting that scoring options such as “this employee can only carry 10–15 kg” or “can only focus attention for 30 min” fail to capture the dynamic nature of work capacity and RTW possibilities.

They also noted that quantitative scoring fails to account for workplace and personal factors that influence work capacity and RTW possibilities. Therefore, most OHPs advocated for removing the quantitative scoring options from the ICF-based instrument in favour of qualitative descriptions, which they felt provided a more contextualized assessment of work capacity. A participant mentioned the following during an interview:

“I think we need to move away from precise assessments in minutes or distance regarding functional limitations. Employers often ask these questions. Recently, I saw a woman with advanced rheumatoid arthritis who worked in education. There was a dispute over which classroom would be her permanent one. The employer asked me to specify her walking distance. But that's not how it works, she feels pain with every step. Sure, in an emergency, she might manage 3 kilometres, but for daily walks to a distant classroom, every meter is too much. We need to focus on what is still possible, but a quantitative assessment is simply both unrealistic and unhelpful.” (Quote from participant 7).

#### Integrating employee’s and employer’s perspectives

OHPs valued the inclusion of questions about employees’ experiences, expectations, and needs regarding sick leave guidance and the RTW process. These insights offer relevant information for identifying suitable job functions, necessary workplace modifications, and potential interventions. However, a key condition for implementation in daily practice was incorporating the employer's perspective alongside the already included employee's perspective. According to OHPs, this would enable them to consider the needs and expectations of both parties when providing RTW advice and guidance.

#### Applying the ICF-based instrument for complex cases of sick-listed employees

OHPs found the instrument particularly useful for complex cases of sick-listed employees.

They emphasized the importance of repeated use throughout the sick leave and RTW process, as it provides a structured overview of changes in work capacity, RTW possibilities, and the advice given.

### Determinants of the socio-political context

#### Applying the ICF-based instrument when it adds value to the RTW process

While OHPs acknowledged the importance of adhering to prescribed milestones outlined in Dutch RTW legislation, they stressed to consider individual circumstances for deciding when to use the ICF-based instrument. They advocated for applying the instrument when it adds value to the RTW process, such as during changes in an employee's work capacity or when the RTW process stagnates. A participant mentioned:

“We should apply the instrument when it adds value to the RTW process, regardless of whether there is an obligation to do so. If I encounter an employee after six weeks of sick leave, and I am certain this person will have permanent limitations, I complete the instrument at that point in time. I then indicate what I expect the future outcomes to be. This is primarily to ensure that the labour expert understands the situation correctly and provides the employee with the right tools” (Quote from participant 2).

### Determinants of the organisation

#### Need for digital implementation of the ICF-based instrument

Most OHPs emphasized the digital integration of the ICF-based instrument into their work systems, such as the electronic patient file they use at the occupational health service, as a key condition for implementation. They believed that a digitally integrated instrument would enhance usability and accessibility by streamlining communication with other OHPs throughout the sick leave and RTW process, while also reducing the time needed to complete and transfer the instrument to other OHPs. A participant noted:

“Completing the ICF-based instrument was a lot of work. But that's partly because nothing has been digitally implemented yet. I expect that once it's fully implemented, personal details would be loaded automatically. For now, I had to enter everything manually, and that was very time-consuming.”

(Quote from participant 9).

## Discussion

This study aimed to assess the feasibility of the ICF-based instrument and multidisciplinary guideline, as well as to explore OHPs’ experiences to support implementation in daily practice.

### Main findings

OHPs highlight that the ICF-based instrument supports a qualitative assessment of work capacity of sick-listed employees, while also capturing relevant workplace and personal factors. In most long-term sick leave cases, the ICF-based instrument was completed during the first-year evaluation, a legally required step under Dutch RTW legislation.^30^ However, OHPs advocated for using the ICF-based instrument when it adds value to the RTW process, regardless of legal obligations. This finding aligns with the socio-political and user domains for implementation outlined by Fleuren et al.,^31^ indicating that although legislation influences the timing of interprofessional collaboration, OHPs are motivated to collaborate beyond legally prescribed moments to better address the specific needs of sick-listed employees.

This highlights a key tension: while OHPs advocate using the ICF-based instrument when most beneficial for a specific sick leave case, they primarily used it at legally mandated moments. This suggests that legally prescribed moments for collaboration may not align with the varying circumstances and needs of individual cases of sick leave, potentially hindering the flexibility needed to effectively guide and support the RTW process.

Another notable finding was that OHPs considered the items of the ICF-based instrument comprehensive to describe work capacity and RTW possibilities and did not suggest incorporating additional ICF-codes, indicating sufficient content validity of the instrument.^34^ Other instruments using the ICF-framework include similar d-codes and have also found these to be important for assessing work capacity and functional limitations.^35^ Additionally, OHPs in this study highlighted that by using d-codes from the activity & participation domain, the ICF-based instrument enabled collaboration between medical and non-medical OHPs.

### Interpretation of the findings

#### A qualitative description of work capacity and RTW possibilities

OHPs highlighted the ICF-based instrument's usability in providing qualitative descriptions of work capacity. They found this particularly valuable for capturing work-related and personal factors that contribute to fluctuations in work capacity and RTW possibilities, enabling more timely and tailored interventions. This finding aligns well with the determinants of the innovation domain^31^ and supports earlier findings emphasizing that work capacity should not be viewed as a fixed or static state but requires a more dynamic approach.^36–39^ Moreover, OHPs emphasized that the ICF-based instrument enables capturing employees’ strengths, rather than focusing solely on limitations. This aligns with the concept of positive health, which encourages viewing individuals beyond their illness and emphasizing their capabilities.^40^ By focusing on employees’ capabilities and strengths, the instrument supports a more contextualized and person-centred assessment of work capacity and RTW possibilities.

#### Removal of quantitative scoring options

Most OHPs recommended removing quantitative scoring options from the ICF-based instrument, advocating to focus on a contextualized and qualitative assessment of work capacity. This finding aligns with previous research emphasizing that work capacity and RTW barriers should be interpreted within the unique context of each individual.^36^ OHPs emphasized that this approach is crucial for effective implementation of the ICF-based instrument, as it allows them to better capture the influence of workplace conditions, social support, and individual coping mechanisms; factors that are known to significantly affect RTW outcomes.^7,10,25,27,35,39^ This is a noteworthy finding, as most existing instruments derived from the ICF are designed with quantitative scoring options to assess work capacity and functional limitations.^10,11,26,35^ Our study adds a new perspective by highlighting that the OHPs preferred using qualitative, contextualized assessment of work capacity over standardized scoring systems. They found that narrative descriptions allowed for a more comprehensive understanding of the employee's situation and better supported interprofessional collaboration. This qualitative approach invites a reconsideration of how the biopsychosocial model can be effectively integrated into occupational health practice to enhance sick leave guidance and the RTW process.

#### How do employees experience their own work capacity and RTW possibilities?

OHPs in this study emphasized the importance of understanding how challenges in work participation not only affect work-related tasks but also employees’ daily lives. This perspective aligns with the concept of the ‘spillover effect,’ which suggests that challenges in one domain, such as work, often extend to other areas of an individual's life, including family dynamics, social interactions, and overall well-being.^37^

By capturing the impact of these challenges on both work and daily life, the ICF-based instrument provides OHPs with a more comprehensive understanding of the employee's challenges and experiences. This finding aligns with previous studies highlighting the importance of improving communication and shared decision-making by focusing on how individuals experience their challenges and health and where they wish to improve.^24,^^40–42^ This also highlights the ICF-based instrument's application as a communication and decision-making aid for sick leave guidance and RTW planning that enhances collaboration among OHPs, employees and employers.

Finally, OHPs highlighted time constraints as a significant barrier, noting that the ICF-based instrument takes longer to complete (15–60 min) than other available tools, particularly in complex cases involving multimorbidity, fluctuating health conditions, psychosocial factors, or conflicting expectations between employees and employers. While these cases required more time, OHPs emphasized that such complexity is characteristic of long-term sick leave and that the ICF-based instrument enables them to comprehensively assess work capacity and identify tailored solutions. To improve usability, they strongly advocated for digital integration of the ICF-based instrument into existing electronic patient file systems, considering this a key condition for implementation. This integration could enhance accessibility, reduce completion time, and strengthen interprofessional collaboration. These findings also align with earlier studies highlighting the need for digital tools to support RTW collaboration.^43,44^

### Methodological strengths & limitations

A key strength of this study was the triangulated mixed methods approach combined with the inclusion of a diverse group of OHPs actively involved in RTW processes. The variety of roles and perspectives contributed to a better understanding of the comprehensibility, usability, and acceptance of the ICF-based instrument and the multidisciplinary guideline in daily practice among different OHPs. The validity of the insights obtained was strengthened by using different kinds of data collection (interviews, reviews of completed ICF-based instruments and focus groups). The insights from semi-structured interviews and reviews of completed ICF-based instruments were validated through member checking and triangulated with findings from focus group discussions.

However, several limitations should be acknowledged. First, although OPs were asked to complete ICF-based instruments for four sick-listed employees, some were not able to complete this task due to time constraints. Second, the ICF-based instrument is designed for repeated use to throughout the course of sick leave and the RTW process, to guide collaboration and monitor change over time. In this study, however, the instrument was supposed to be only completed once per employee. As a result, we were unable to evaluate its longitudinal applicability. Future research should explore the feasibility and added value of repeated application over the course of sick leave and RTW.

### Implications for practice, policy and research

#### Occupational health practice

The ICF-based instrument enables OHPs to capture both the strengths and limitations of employees, along with relevant workplace and personal factors, allowing for more tailored guidance to the individual's context. By considering contextual factors, strengths and prior work experience, the instrument supports a more person-centred assessment of work capacity and possibilities for RTW. This approach is particularly valuable when a return to the original job is no longer feasible and alternative work options need to be considered.

#### For policy and government regulators

OHPs in this study advocated for using the ICF-based instrument when it adds value to the RTW process, such as in complex cases or when RTW is stagnating. Rather than limiting its use to fixed legal moments only, applying the instrument more strategically allows for better alignment with the unique context of each long-term sick leave case. Our findings suggest that sick leave guidance and RTW collaboration in the Netherlands could benefit from regulations that emphasize the importance of timing moments of collaboration for what is most beneficial for the specific RTW process.

#### For academia and researchers

This study contributes to implementation science in occupational health by demonstrating how the ICF framework can support collaboration, among medical and non-medical OHPs and between OHPs, employees, and employers. It opens avenues for further research into the role of qualitative tools in enhancing interprofessional collaboration.

## Conclusion

This study highlights the potential of implementing the ICF-based instrument along with an accompanying multidisciplinary guideline in the daily practice of OHPs to enhance interprofessional collaboration during sick leave and the RTW process. OHPs reported that the instrument supports a qualitative assessment of both the strengths and limitations of sick-listed employees, while also capturing relevant workplace and personal factors. This facilitates more tailored, person-centred guidance and collaboration. By using d-codes from the ICF-framework, the instrument facilitates communication between medical and non-medical OHPs and promotes shared decision-making by incorporating the perspectives of both employees and employers. Strategic use of the instrument, at moments when it adds value to the RTW process, was identified as essential for successful implementation. Future research should examine its digital integration into occupational health systems.

## Appendix 1. Overview of the ICF-based instrument and the multidisciplinary guideline

**Components of the ICF-based instrument:**

**Domain 1: Work Factors**

- **Psychosocial Workload**
  ○ Time pressure
  ○ Responsibility of the worker
  ○ Structure of tasks
- **Workplace Environment**
  ○ Noise
  ○ Elevated personal risk
  ○ Substances, light, and temperature at the workplace
  ○ Ergonomics of the workplace
- **Work Times**
  ○ Work hours and scheduling demands

**Domain 2: Activities and Participation (d-codes from the ICF-framework)**

- **Personal Functioning**
  ○ d159 Basic learning (other specified and unspecified)
  ○ d160 Focusing attention
  ○ d175 Solving problems
  ○ d177 Making decisions
  ○ d220 Undertaking multiple tasks
  ○ d240 Handling stress and other psychological demands
- **Social Functioning**
  ○ d110 Watching
  ○ d115 Listening
  ○ d120 Other purposeful sensing
  ○ d330 Speaking
  ○ d470/d475 Using transportation / Driving
  ○ d720 Complex interpersonal interactions
  ○ d740 Formal relationships
- **Physical Functioning**
  ○ d450 Walking
  ○ d451 Going up and down stairs
  ○ d430 Lifting and carrying objects
  ○ d440 Fine hand use
  ○ d445 Hand and arm use
  ○ d410 Changing basic body position
  ○ d415 Maintaining body position

**Domain 3: Personal Factors**

○ Personal factors

**Examples of recommendations in the multidisciplinary guideline:**

Regarding communication between OHPs, it is recommended that:

- The OP and the IP consult with each other when clarification is needed regarding the work capacity or the RTW possibilities of a sick-listed employee.
- The OP, LE and IP contact each other if they disagree or when RTW is stagnating

## Appendix 2. Interview questions with occupational physicians

1. How did you experience working with the ICF-based instrument?
  ○ What went well? What went less well?
2. Is the ICF-based instrument effective for describing work capacity?
  ○ How was your experience using the instrument to describe work capacity? What did you miss? Can you give examples?
3. Can you adequately describe RTW possibilities using the ICF-based instrument?
  ○ What did you miss? Can you give examples?
4. How much time did it take to complete an ICF-based instrument?
  ○ Did it take more time compared to other instruments?
5. Do the components of the ICF-based instrument align with your usual working methods?
  ○ Why do they align/do not align?
6. Are the components of the ICF-based instrument structured clearly and logically?
  ○ What makes the components clear or unclear?
7. What are the key advantages and disadvantages of working with the ICF-based instrument?
  ○ Can you give examples? How does the ICF-based instrument compare to the tools you typically use?
8. On a scale of 0–10, how usable is the ICF-based instrument overall?
  ○ (0 = not usable, 10 = highly usable)
9. On a scale of 0–10, how comprehensible is the ICF-based instrument overall?
  ○ (0 = not comprehensive, 10 = highly comprehensive)
10. On a scale of 0–10, how satisfied are you with the ICF-based instrument?
  ○ (0 = not satisfied, 10 = highly satisfied)

The ICF-based instrument includes an open-ended question about personal factors that may play a role in work capacity or RTW possibilities.

11. Did you fill out anything in the item regarding personal factors? Were you able to answer adequately?
12. Would you prefer to report on personal factors in the ICF-based instrument, if so, in what manner?

**Questions about using the multidisciplinary guideline:**

13. Have you consulted the multidisciplinary guideline?
  - If yes, for what? Was it useful? What made the guideline clear or unclear?
14. For what purpose did you apply the ICF-based instrument?
  - How did it go? Do you see any opportunities to use the ICF-based instrument for other purposes?
15. Does the multidisciplinary guideline provide sufficient guidance/information for completing the instrument?
16. Do you think the ICF-based instrument is useful for drafting/adjusting the RTW plan?

You may have coordinated with the employer, employee, or other OHPs, by means of the completed instrument. We are curious about your experiences.

17. With which stakeholders did you share the completed ICF-based instrument?
  - How did you experience this? How did you proceed (e.g., verbally, in writing, by phone, via email)? Can you provide examples? Did other stakeholders have questions about the ICF-based instrument?
18. Did the collaborative process change due to the use of the ICF-based instrument?
  - Can you elaborate on how it went? What were the advantages and disadvantages?
19. Do you expect that collaboration with other professionals will change because of using the ICF-based instrument?
  - Can you explain this? Would you like anything to change in the collaboration
20. Do you expect that your communication with the employee and employer will change due to the use of the ICF-based instrument?
  - Can you explain? Are the opportunities for improving communication? Are the risks?

## Appendix 3. Case review questionnaire

**Comprehensibility**

1. Can you determine the key areas of concern for the client's capacity from the ICF-based instrument?
2. Can you describe the main areas of attention for the client?
3. Is it clear to you what the client's return to work possibilities are?
4. Can you describe the client's return to work possibilities?
5. Does the completed ICF-based instrument provide a clearer indication of work capacity and return to work possibilities compared to the instruments you normally use/receive? (What do you find clearer/less clear about the ICF-based instrument? Compared to which instrument?)
6. On a scale of 0–10, how comprehensible is the information filled in the ICF-based instrument overall? (0 = not understandable, 10 = completely understandable) (Can you explain what makes the completed ICF-based instrument understandable/not understandable?)

**Usability of the ICF-based Instrument**

7. Is the ICF-based instrument useful for job tasks? (in what way?)
8. Is the ICF-based instrument useful for providing return to work advice? (Can you elaborate?)
9. Is the ICF-based instrument useful for assessing suitable tasks and functions for the employee? (Can you elaborate?)
10. Is the ICF-based instrument useful for other purposes? If so, can you specify which?
11. On a scale of 0–10, how useful is the completed ICF-based instrument for your work? (0 = not useful, 10 = very useful) (Can you explain what you find useful/not useful?)
12. Is the completed ICF-based instrument more useful than the instruments you normally use/receive? (Can you elaborate?)

**Acceptance**

13. On a scale of 0–10, how satisfied are you with this completed ICF-based instrument? (0 = not satisfied, 10 = very satisfied) (Can you explain what you are satisfied/not satisfied with?)

**Questions about using the multidisciplinary guideline**

14. Have you consulted the guideline? (If so, for what purpose? Was it useful? What makes the guide clear/not clear?)
15. For what purpose did you apply the ICF-based instrument? (Do you see any additional possibilities for using the ICF-based instrument?)
16. Does the guideline provide sufficient instruction/information for using and interpreting the ICF-based instrument?

**Suggestions for improving the ICF-based instrument**

17. Do you have any suggestions for improving the use of the ICF-based instrument? Or for the (completion instructions in the) ICF-based guideline?

## Appendix 4. Focus group guide

**Sharing experiences of working with the ICF-based instrument**

To start, I we would like to ask all participants to briefly share their experience working with or interpreting the ICF-based instrument. OHPs will be asked whether they recognize the insights presented in the handouts. They will also have the opportunity to respond to specific points in the handouts.

**A. Involving employees and employers in the ICF-based instrument**

During the feasibility study, it was frequently mentioned that the added value of the ICF-based instrument could be to involve both the employee and employer more in the RTW process. Several ideas were proposed by OHPs to achieve this goal:

- Add an extra section for the OP where the information of the ICF-based instrument is summarized in an understandable way for the employee/employer (formulating conditions for RTW)
- Ask the employees and employers several questions in the ICF-based instrument: “What do you consider important points to discuss with the OP? What is going well? What are your main obstacles for returning to work? What do you need to overcome them?”

**B. Shifting the focus from limitations to possibilities**

Most OHPs in this study mentioned that the ICF-based instrument still focuses too much on limitations rather than possibilities, therefore, the following ideas were proposed:

- OPs should primarily indicate what is still possible.
- Avoid quantitative scoring options in the instrument

**C. Clarifying areas of attention and return to work possibilities**

Several suggestions were made by OHPs that could lead to more consistent interpretations of work capacity and return to work possibilities:

- Provide example scenarios illustrating how key areas manifest in daily life/work.
- Indicate timeframes for how long areas of attention are expected to persist.

**E. Conditions for using the ICF-based Instrument**

Another key goal of the study is to identify the conditions necessary for OHPs to effectively use the ICF-based instrument. What are important conditions to adopt and apply the ICF-based instrument and the multidisciplinary guideline in daily practice?

**F. Closing with suggestions and recommendations**

Are there additional ideas, suggestions or recommendations that have not yet been discussed?

## Appendix 5. Final coding scheme from interviews

**Table:**

Code System Feasibility study ICF-based instrument	Frequency
Code System	478
Experience of the occupational physician	
Experience as occupational physician	10
Experience with the ICF-based instrument	14
MIDI Innovation determinants	
MIDI Inn determinants use of innovation\social security agency (politics/legislation)	7
MIDI Inn determinants use of innovation\ICF framework & scientific basis	12
MIDI Inn determinants use of innovation\Usability of the ICF-based instrument	10
MIDI Inn determinants use of innovation\Usability for assessing RTW efforts	8
MIDI Inn determinants use of innovation\Usability when RTW is not possible	9
MIDI Inn determinants use of innovation\Usability in providing RTW advice	12
MIDI Inn determinants use of innovation\Usability for drafting the problem analysis	3
MIDI Inn determinants use of innovation\Usability for the first year evaluation	11
MIDI Inn determinants use of innovation\Clear and logical structure	10
MIDI Inn determinants use of innovation\Describing personal factors	8
MIDI Inn determinants use of innovation\Work factors & psychosocial workload	7
MIDI Inn determinants use of innovation\ICF-based instrument offers a common language	9
MIDI Inn determinants use of innovation\Value of information about personal and social factors	13
MIDI Inn determinants use of innovation\ICF-based instruments provides more room for explanations	9
MIDI Inn determinants use of innovation\Employee's perspective in the ICF-based instrument	13
MIDI Inn determinants use of innovation\Dynamic representation instead of snapshot of RTW	19
MIDI Inn determinants use of innovation\Not fit for early stages of RTW	13
MIDI Organizational determinants	
MIDI Org determinants for use from the organization\Support from professional associations needed	15
MIDI Org determinants for use from the organization\Fits with current working methods	8
MIDI Org determinants for use from the organization\Time to complete the ICF-based instrument	8
MIDI User determinants	
MIDI User determinants/Comprehensibility of the ICF-based instrument	17
MIDI User determinants/Usability of the ICF-based instrument	12
MIDI User determinants/Satisfaction with the ICF-based instrument	23
MIDI User determinants/Completing the ICF-based instrument takes much time	14
MIDI socio-political determinants	
MIDI socio-political determinants\Use to describe RTW possibilities	18
MIDI socio-political determinants\Use to describe work capacity	9
MIDI socio-political determinants\Against describing the areas of attention quantitively	12
MIDI socio-political determinants\Purpose of the ICF-based instrument is RTW of the employee	23
MIDI socio-political determinants \Occupational physician must have freedom when to use the instrument	10
MIDI socio-political determinants\Differences and similarities with other instruments	14
MIDI socio-political determinants\the ICF-based instrument can provide guidance and support	11
Coordination with other stakeholders	
Coordination with other stakeholders\With other occupational physicians	4
Coordination with other stakeholders\With the Employee	21
Coordination with other stakeholders\With the Employer	17
Coordination with other stakeholders\With the Labour expert	10
Coordination with other stakeholders\Expectation of coordination change	13
